# The arginine repressor ArgR controls virulence factors of adherent-invasive *Escherichia coli* strains

**DOI:** 10.3389/fcimb.2026.1941024

**Published:** 2026-10-12

**Authors:** Queralt Bonet-Rossinyol, Margarita Martinez-Medina, Llorenç Fernández-Coll

**Affiliations:** Microbiology of Intestinal Diseases, Biology Department, Universitat de Girona, Girona, Spain

**Keywords:** Adherent-invasive *Escherichia* coli (AIEC), arginine biosynthesis, ArgR, biofilm formation, Crohn’s disease, invasion, motility, transcriptomics

## Abstract

**Introduction:**

Adherent-invasive Escherichia coli (AIEC) has been related to the physiopathology of Crohn’s disease, yet its pathogenic mechanisms are not completely understood. Arginine biosynthesis genes are upregulated in AIEC strains compared to non-AIEC strains present in the supernatant of infected intestinal epithelial cells, suggesting that arginine biosynthesis may be involved in AIEC pathogenicity.

**Methods:**

To test this hypothesis, the argR gene, encoding the transcriptional repressor ArgR, was deleted in the AIEC strains AIEC16 and LF82, and in E. coli Nissle 1917 and K-12 C600, leading to increased expression of arginine biosynthesis genes. Phenotypic assays and transcriptomic analyses were conducted to assess differences between mutants (ΔargR) and wild-type (WT) strains.

**Results:**

The absence of ArgR led to a more invasive and motile AIEC LF82 strain. No differences in invasion were found for AIEC16 and the mutation led to reduced motility and biofilm formation in this strain. Transcriptomic analysis was in line with the phenotypic observations and further revealed that ArgR regulates the expression of multiple virulence-associated genes. Acid resistance genes (gad operon) were upregulated in ΔargR strains, indicating a repressive role of ArgR. Conversely, genes involved in adhesion, lipopolysaccharide biosynthesis, and iron uptake were downregulated in mutant strains, suggesting that ArgR functions as an activator for these pathways.

**Discussion:**

These findings highlight ArgR as a potential modulator of multiple virulence traits, influencing the invasive and adaptive capabilities of AIEC.

## 1 Introduction

Adherent-invasive *Escherichia coli* (AIEC) has been associated with Crohn’s disease (CD), a chronic inflammatory bowel disease of undetermined etiology (Darfeuille-Michaud et al., 2004; Baumgart et al., 2007; Eaves-Pyles et al., 2008; Martinez-Medina et al., 2009). AIEC strains are characterized by their ability to adhere to and invade intestinal epithelial cells, and to survive and replicate within macrophages without inducing apoptosis and increasing TNF-α secretion (Boudeau et al., 1999; Glasser et al., 2001). Moreover, AIEC lack specific virulence genes typically found in enteropathogenic *E. coli* strains and are genetically close to extra-intestinal pathogenic *E. coli* (ExPEC) (Martinez-Medina et al., 2009; Miquel et al., 2010; Nash et al., 2010). Several studies have reported a higher prevalence of AIEC in CD patients compared to healthy subjects (Khan et al., 2021; Nadalian et al., 2021), and AIEC colonization has been related with several of the pathophysiologic characteristics of the inflamed gut in CD, such as inflammation and granuloma formation (Meconi et al., 2007; Mansour et al., 2023). Furthermore, AIEC strains can adapt to the inflamed environment and outcompete other bacteria (Mansour et al., 2023). Altogether suggest a potential role of AIEC in the development of the disease.

However, the molecular mechanisms involved in AIEC pathogenicity are not fully understood, and the genetic differences between AIEC strains and other *E. coli* strains, such as ExPEC or commensal, are not clear. Therefore, no molecular markers are available for its identification (Camprubí-Font and Martinez-Medina, 2020).

In a previous study, we suggested that the differences between AIEC and non-AIEC phenotypes are not driven by the genetic content but by gene expression changes. In a collection of 14 AIEC and 23 non-AIEC strains, genes involved in the arginine biosynthesis pathway were differently expressed during infection of intestinal epithelial cells. These genes were found overexpressed in the supernatant fraction (containing free bacteria) of AIEC strains compared to non-AIEC strains (Bonet-Rossinyol et al., 2023).

In *E. coli*, the arginine biosynthesis pathway consists of eight sequential enzymatic steps and uses glutamate as a substrate (Charlier and Bervoets, 2019). The genes involved in this pathway are distributed across eight operons that are located throughout the genome. Among them, the genes *argB, argC* and *argH* form a single transcriptional unit, while *argA, argD, argE, argF, argG, argI*, and *gabT* are transcribed independently (Gigot et al., 1980). All these genes are under regulation of ArgR (Maas, 1964), which acts as a repressor for arginine transport and synthesis (Cho et al., 2015), and as an activator of genes involved in arginine degradation (Kiupakis and Reitzer, 2002). The activity of ArgR depends on the presence of arginine, that binds to the hexameric ArgR and promotes a conformation change that will allow it to bind to DNA (Wang et al., 1998; Charlier and Bervoets, 2019).

Arginine may play an important role in *E. coli* pathogenicity. It is known to be involved in the arginine decarboxylase acid resistance system (Adi), where the arginine decarboxylase AdiA converts arginine into agmatine while consuming a proton. This mechanism might enhance AIEC’s survival in acidic conditions, such as intracellular endocytic vacuoles or phagolysosomes as well as in the gastrointestinal tract (Brameyer et al., 2022). Moreover, it has been reported that *E. coli* can use arginine to synthesize putrescine and spermidine, two polyamines that can increase protein synthesis at the translation level (Igarashi and Kashiwagi, 2018; Charlier and Bervoets, 2019) and induce the expression of genes related to oxidative stress response (Tkachenko and Nesterova, 2003).

In a high-throughput fitness screening (Elhenawy et al., 2021), different genes involved in arginine biosynthesis were identified to be essential for AIEC’s fitness in mice intestines, among them *argR*. Moreover, they determined that the expression of several arginine biosynthetic genes is increased in bacteria grown in the *in vivo* model (mouse) compared to *in vitro* cultures (Elhenawy et al., 2021). Additionally, a comparative transcriptomic study conducted during cell culture infection with Shiga toxin *Escherichia coli* (STEC) and commensal *E. coli* strains revealed that arginine biosynthesis genes were upregulated in adhered STEC strains compared to their planktonic counterparts, a pattern not observed in commensal strains (Harrison et al., 2020). In studies comparing the most abundant biosynthetic pathways expressed in CD patients and healthy subjects, the arginine biosynthesis pathway was the one with higher relative abundance in CD patients compared with healthy subjects (Hu et al., 2021). Collectively, these findings suggest arginine biosynthetic genes may play an important role in AIEC colonization and pathogenicity.

Our study aims to elucidate the role of arginine in AIEC pathogenicity using Δ*argR* mutant strains. Given that arginine biosynthesis genes are distributed across multiple operons in the *E. coli* genome, ArgR was deleted to achieve an overexpression of all the genes within this regulon. The physiological and pathogenic characteristics of both wild-type (WT) and mutant strains were assessed to determine the conditions or processes influenced by arginine. Additionally, a comparative transcriptomic analysis between WT and Δ*argR* strains was performed to identify other ArgR-regulated genes potentially linked to the AIEC phenotype.

## 2 Materials and methods

### 2.1 Strains of study, plasmids and primers

The strains and plasmids used in this study are listed in **Supplementary Table 1**. Two AIEC (LF82 and AIEC16) and two non-AIEC strains (*E. coli* Nissle 1917 and *E. coli* K-12 C600) were used to study the role of arginine biosynthesis genes into the AIEC phenotype by generating Δ*argR* mutants. LF82 and *E. coli* K-12 C600 strains were kindly provided by Dr. Nicolas Barnich, *E. coli* Nissle 1917 was cultivated from Mutaflor^®^, and AIEC16 was isolated in our laboratory (Martinez-Medina et al., 2009). Deletion of *argR* was performed through the λ Red recombinase protocol (Datsenko and Wanner, 2000). Kanamycin-resistant gene with homology flanks was amplified from pKD4 with primers argR-FW and argR-RV. Then it was introduced into the chromosome through the λ Red recombinase expressed from pKD46. Kanamycin-resistant clones were selected in a 50 µg/ml Kanamycin agar and verified by PCR with the primer pairs arg1 and arg2 (**Supplementary Table 2**).

All the primers used in this study (**Supplementary Table 2**) were manually designed based on the genomic sequences of the strains selected for mutant generation. The absence of secondary structures was verified for each primer pair using the NetPrimer software (PRIMER Biosoft International, Palo Alto, CA).

### 2.2 Quantification of gene expression by RT-qPCR

We quantified the expression levels of three genes involved in the arginine biosynthesis pathway (*argB, argC*, and *argH*) in the Δ*argR* and the WT strains. mRNA was obtained from overnight cultures with 3 mL of Luria-Bertani (LB) broth for each strain. TRIzol (Invitrogen, USA) was used following manufacturer’s instructions, solubilizing the pellet with 40 µL of TE buffer in the last step. Subsequently, a DNase TURBO treatment (ThermoFisher, USA) was applied according to the manufacturer’s instructions. mRNA concentration was quantified with NanoDrop ND-1000 spectrophotometer (NanoDrop Technologies, USA). Then, cDNA was synthetized using High-Capacity cDNA Reverse Transcription kit (Applied Biosystems, USA), following manufacturer’s instructions.

To quantify gene expression, 8 µl of a 1/100 dilution of cDNA were mixed with 10 µl of SYBR^®^ Green PCR Master Mix (Applied Biosystems, USA), 1 µl of forward primer and 1 µl of reverse primer (**Supplementary Table 2**). At this point, *gapA* was selected as housekeeping gene (Jandu et al., 2009). The 96-well plate was introduced to the 7500 Real Time PCR System using the 7500 System SDS Software (Applied Biosystems, USA) with the Standard Curve option. Following an initial denaturation step at 95°C for 10 minutes, amplification was performed for 40 cycles of 15 seconds at 95°C and 1 minute at 60°C. Fold change was calculated using the ΔCt method (Schmittgen and Livak, 2008).

### 2.3 Adhesion and invasion assay

Adhesion and invasion of Intestine-407 (ATCC CCL-6™) intestinal epithelial cell line was performed with a multiplicity of infection (MOI) of 10 as previously described (Boudeau et al., 1999). It is considered that the strain presents the adherent-invasive phenotype if the adhesion index is higher than 1 bacteria/cell and the invasion index is higher than 0.1% (Darfeuille-Michaud et al., 2004).

### 2.4 Survival/replication within macrophages

Survival and replication within macrophages was assessed using the J774A.1 (ATCC TIB67) macrophage cell line as previously described (Glasser et al., 2001). Strains with a survival rate above 100% were considered to survive, while those with a rate above 200% were considered able to replicate inside of macrophages.

### 2.5 Motility

Motility agar plates were prepared by adding 10 g of tryptone, 5 g of NaCl and 2.5 g of bacteriological agar in 1 L of distilled water (Partridge and Harshey, 2020). After 18 hours, 5 µl of an over-night bacterial culture was inoculated. The diameter of the halo was measured after 8 hours at 37°C to determine the swimming motility.

### 2.6 Acid resistance assay

In this assay, 100 µl of a 10^5^ colony forming units (cfu)/ml bacterial suspension were inoculated in 5 ml of Ringer’s solution at pH 7, and in 5 ml of citrate buffer at pH 4, 3 and 2. Samples were plated on LB agar plates to determine cfu at time 0 and after 1 hour at 37°C. Finally, the survival percentage at 1 hour relative to the initial count was calculated.

### 2.7 Biofilm formation

Biofilm formation was determined as previously described by (Tajuelo et al., 2023). Briefly, bacterial suspensions of 10^8^ cfu/ml in CFA medium (10 g/l casamino acids, 1.5 g/l yeast extract, 50 mg/l NgSO_4_, and 5 mg/l MnCl) was prepared for each strain to be tested, and 200 µl were added to round-bottom 96-well plates. Three wells containing only CFA medium were used to create the blank. After an incubation of 24 h at 37°C, the absorbance at 600nm was measured to determine bacterial growth using the SynergyTM 4 plate reader (BioTek, USA). The supernatant from each well was discarded, and 200 µl of a 1% crystal violet solution was added to each well. They were then incubated at room temperature for 15 minutes, followed by three washes with 250µl of PBS in each well. Finally, 200 µl of 70% ethanol was added to solubilize the crystal violet, and the absorbance was measured at 596nm. Biofilm production was quantified normalizing the absorbance at 596nm to the absorbance at 600nm.

### 2.8 Comparative transcriptomics between Δ*argR* and WT strains

RNA was isolated from overnight cultures grown in LB broth at 37°C in triplicate using TRIzol (Invitrogen, USA), and remaining genomic DNA was eliminated with the TURBO DNase kit (ThermoFisher, USA). Ribosomal RNA was depleted using the Ribo-Zero Plus rRNA Depletion kit (Illumina, USA), and RNA was precipitated using the sodium acetate method (Maniatis et al., 1982). RNA quantity and quality were assessed with the Qubit Fluorometer using the RNA HS Assay kit (Thermo Scientific, USA), and the Agilent RNA 6000 Nano Chip Bioanalyzer (Agilent, USA) for total prokaryotic RNA.

Libraries were built with the NEBNext^®^ Ultra TM II Directional RNA Library Prep kit (Illumina, EUA) without fragmentation. Then, the samples were sequenced with NovaSeq PE150 (Illumina, USA) with a sequencing depth of 10 million reads. The quality of the FASTQ files was assessed with the software FastQC v0.11.5, and the output was summarized using MultiQC (Ewels et al., 2016). Trimmomatic v0.39 software (Bolger et al., 2014) was used to remove the adapters with the ILLUMINACLIP option, and low-quality regions with the HEADCROP:12 and TRAILING:25 options. Sequences shorter than 40 bp were discarded using MINLEN:40. The reads were aligned to the reference genome of *Escherichia coli* LF82 (GenBank access: GCA_021398935.1) with STAR 2.7.8a (Dobin et al., 2013). Read counts were computed using htseq-counts v0.13.5 (Anders et al., 2015) and normalized and filtered with the Trimmed Mean of M-values method) (Robinson and Oshlack, 2010). The Bioconductor packages NOISeq and NOISeqBIO were used to conduct a differential expression analysis (Tarazona et al., 2011), and the probability threshold was set to q = 0.95. Gene expression levels were presented as fragments per kilobase of transcript per million mapped reads (FPKM). For each gene, fold change was calculated by dividing the FPKM values of the gene obtained in Δ*argR* by those obtained in WT. Only those genes with fold change ≥ 2 were considered differentially expressed genes (DEGs). Then, log_2_ was applied to fold change values. Therefore, positive log_2_ fold change values indicated upregulated genes in the mutant strain (repressed by ArgR), while negative log_2_ fold change values indicated downregulated genes in the mutant strain (activated by ArgR).

DEGs were classified according to their function using the database of Clusters of Orthologous Genes (COGs) (Tatusov et al., 2000), and those involved in the arginine transport, biosynthesis and degradation were identified. Furthermore, bibliographic research was used to highlight those DEGs involved in the pathogenicity of AIEC.

## 3 Results

### 3.1 Quantification of *arg* genes expression for mutant validation

In this study, we used the reference AIEC strain LF82 and the clinical isolate AIEC16, both previously isolated from Crohn’s disease patients. The probiotic strain Nissle 1917 and the laboratory strain K-12 C600 were used as non-AIEC strains. Isogenic Δ*argR*::Km^R^ were constructed for each strain. No significant differences were observed in their growth rate in LB. Given that ArgR functions as a repressor of arginine biosynthesis genes, we expected its deletion to result in increased expression of these genes. To confirm this, we quantified the expression of three genes in this pathway (*argB*, *argC* and *argH*) by RT-qPCR in overnight cultures. As expected, all four Δ*argR* mutants exhibited increased expression of *argB*, *argC*, and *argH* compared to their respective WT strains (**Table 1**).

**Table 1:** Quantification of *arg* genes expression in Δ*argR* and WT strains by RT-qPCR.

Strain	*argB*	*argC*	*argH*
LF82	147.1 ± 55.13	165.4 ± 82.29	56.49 ± 12.12
AIEC16	17.24 ± 9.47	14.47 ± 9.17	13.89 ± 2.24
K-12	461.77 ± 118.28	805.9 ± 165.71	78.59 ± 14.54
Nissle	8.78 ± 0.32	7.12 ± 0.84	9.28 ± 1.31

Fold change values are shown for Δ*argR* using the corresponding WT as the reference strains. A value greater than 1 indicates gene overexpression in the mutant strain.

### 3.2 Impact of *argR* deletion on the phenotypic characteristics of AIEC and non-AIEC strains

To assess the general impact of *argR* deletion, we evaluated bacterial motility and biofilm formation (**Figure 1**). Swimming motility was determined by measuring colony diameter after 8 hours at 37 °C on motility plates (**Figure 1A**). Significant differences were only observed in AIEC strains. The LF82 Δ*argR* strain exhibited increased motility (40 mm diameter) compared to its WT counterpart (27.8 mm diameter) (p = 0.0049). Conversely, the AIEC16 Δ*argR* strain showed decreased motility, with a colony diameter of 24.5 mm compared to the 35 mm in the AIEC16 WT strain (p = 0.0050). For biofilm formation, a significant decrease was observed only in the AIEC16 strain (**Figure 1B**), while no significant changes were observed in the other three strains.

**Figure 1:**
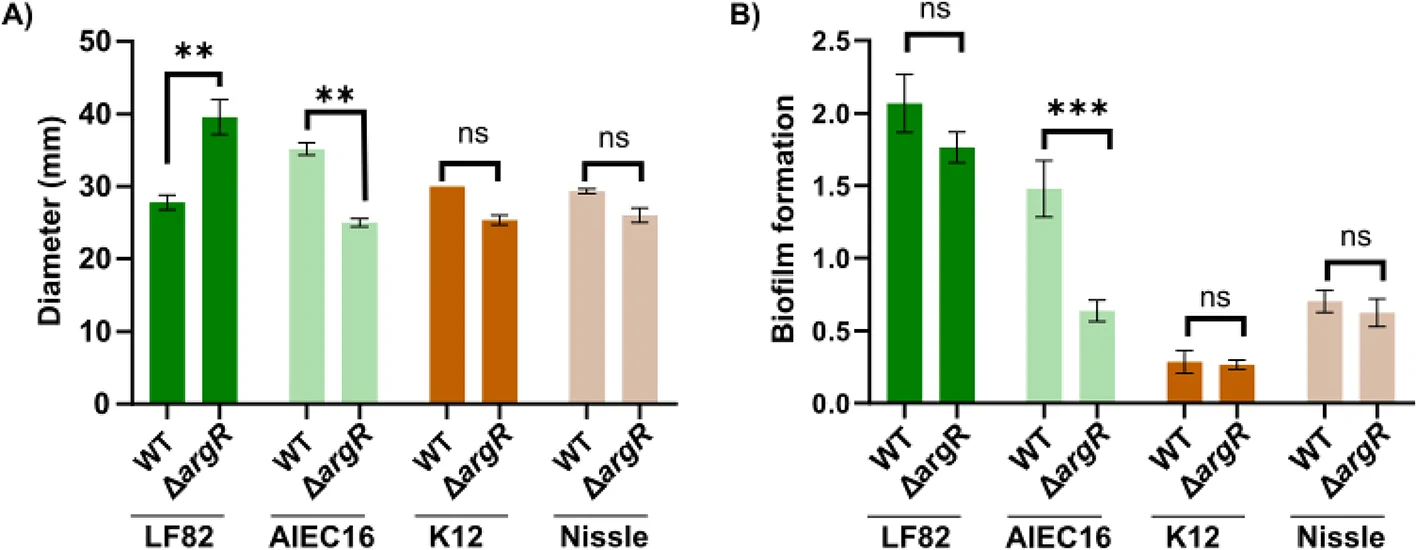
Effects of ArgR deletion on the swimming and biofilm formation phenotypes. **(A)** Swimming motility assay. Diameters of the colonies are shown for each strain with three biological replicates. Mann-Whitney test was used to assess statistical significance. **(B)** Biofilm formation assay. Biofilm production was quantified normalizing the absorbance at 596nm to the absorbance at 600nm from three biological replicates. T-student test was used to assess statistical significance. ns P>0.05; *p ≤ 0.05; **p ≤ 0.005; ***p ≤ 0.001.

Considering that arginine can be used to survive to acid conditions (Adi system), the ability of Δ*argR* strains and WT strains to survive under acidic conditions was evaluated. The strains were subjected to pH 2, 3, and 4 citric buffer and survival was measured after one-hour incubation. No statistically significant differences were found between the mutant and WT strains. All strains could survive above 56% under both pH 4 and pH 3 conditions, while under pH 2, survival decreased to 14-53% (**Supplementary Figure 1**).

The phenotypic traits that characterize and define the AIEC pathotype were also assessed, including the ability to adhere to and invade epithelial intestinal cells, as well as to survive and replicate within macrophages (Boudeau et al., 1999; Glasser et al., 2001). Despite no significant differences were observed regarding adhesion levels (**Figure 2A**), the invasion index of LF82 Δ*argR* was increased by 1.73 in comparison to the WT strain (p = 0.038), and by 2.16 in the case of Nissle Δ*argR* (p = 0.036) (**Figure 2B**). No significant differences were observed between Δ*argR* and their respective WT strains in ability to survive and replicate inside J774 macrophages (**Figure 2C**).

**Figure 2:**
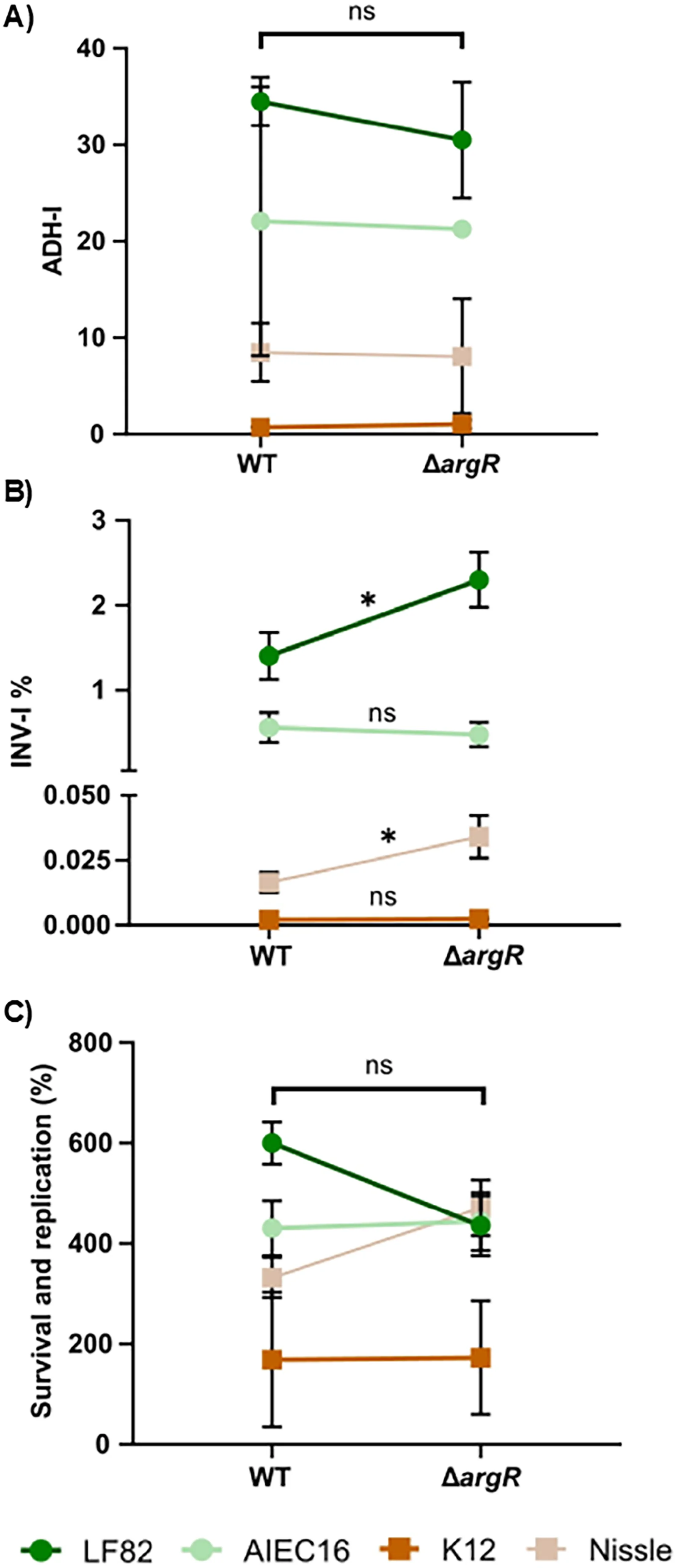
Effects of ArgR deletion on the adhesion to and invasion of intestinal epithelial cells, and survival/replication within macrophages. **(A)** Adhesion index in I-407 (bacteria/cell). **(B)** Invasion index in I-407 (% internalized bacteria at 4h p.i.). **(C)** Survival and replication index in macrophages (% bacteria at 24h p.i./bacteria at 1h p.i.). Paired T-test was performed to minimize the variability among assays. ns P>0.05; *p-value < 0.05.

### 3.3 Comparative transcriptomics between Δ*argR* and WT strains

To identify genes potentially regulated by ArgR, mRNA sequencing was performed on LF82, AIEC16, Nissle, K-12, along with their respective Δ*argR* mutants, growing in liquid LB medium cultures. This analysis yielded between 9.5 to 21 million reads per sample, and the alignment percentage to the reference genome of the LF82 strain was above 87.9% in all cases (**Supplementary Table 3**). Comparative transcriptomics were performed between Δ*argR* mutants and their respective WT strains using a probability threshold (q) of 0.95 and a fold-change threshold of ± 2 to be considered differentially expressed genes (DEGs). Genes found upregulated in the mutant strain (fold-change ≥ 2) are repressed by ArgR (**Supplementary Table 4**), while genes found downregulated in the mutant strain (fold-change ≤ -2) are activated by ArgR (**Supplementary Table 5**).

Overall, 576 differences of expression were detected among the four comparisons performed (**Table 2**). A major number of genes was found differently expressed in absence of Δ*argR* in the strains Nissle and AIEC16 (274 and 178 genes respectively), while in LF82 we detected 89 DEGs and in K-12 only 35. ArgR acts mainly as a repressor in LF82, AIEC16 and K-12, finding a major number of genes upregulated than downregulated in most of the strains (80%, 66% and 97% of upregulated genes respectively), while it seems to have a more prominent role as activator in Nissle (67% of genes were downregulated in the mutant strain).

**Table 2:** Number of DEGs detected in the comparative transcriptomics analyses between Δ*argR* and WT strains.

Strain	DEGs Fold change > 2		
	Downregulated in Δ*argR*	Upregulated in Δ*argR*	Total
LF82	18	71	89
AIEC16	60	118	178
K-12	1	34	35
Nissle	184	90	274

Among the genes commonly affected by ArgR in all four strains, 14 were upregulated (**Figure 3A**), while none was found downregulated (**Figure 3B**). Of those 14 genes, 10 were part of the arginine biosynthesis pathway (a*rgA, argB, argC, argD, argE, argF, argG, argH, carA*, and *carB*), three were involved in arginine transport (*argO, artJ*, and *ydgI*), and one has a poorly characterized function (*yhcC*). Moreover, those genes involved in arginine biosynthesis pathway presented the greatest upregulation in mutant strains, and those with the greatest downregulation were related with arginine degradation (**Figure 3C**; **Supplementary Table 6**). This confirms the mutation generated allows the overexpression of the arginine biosynthesis pathway.

**Figure 3:**
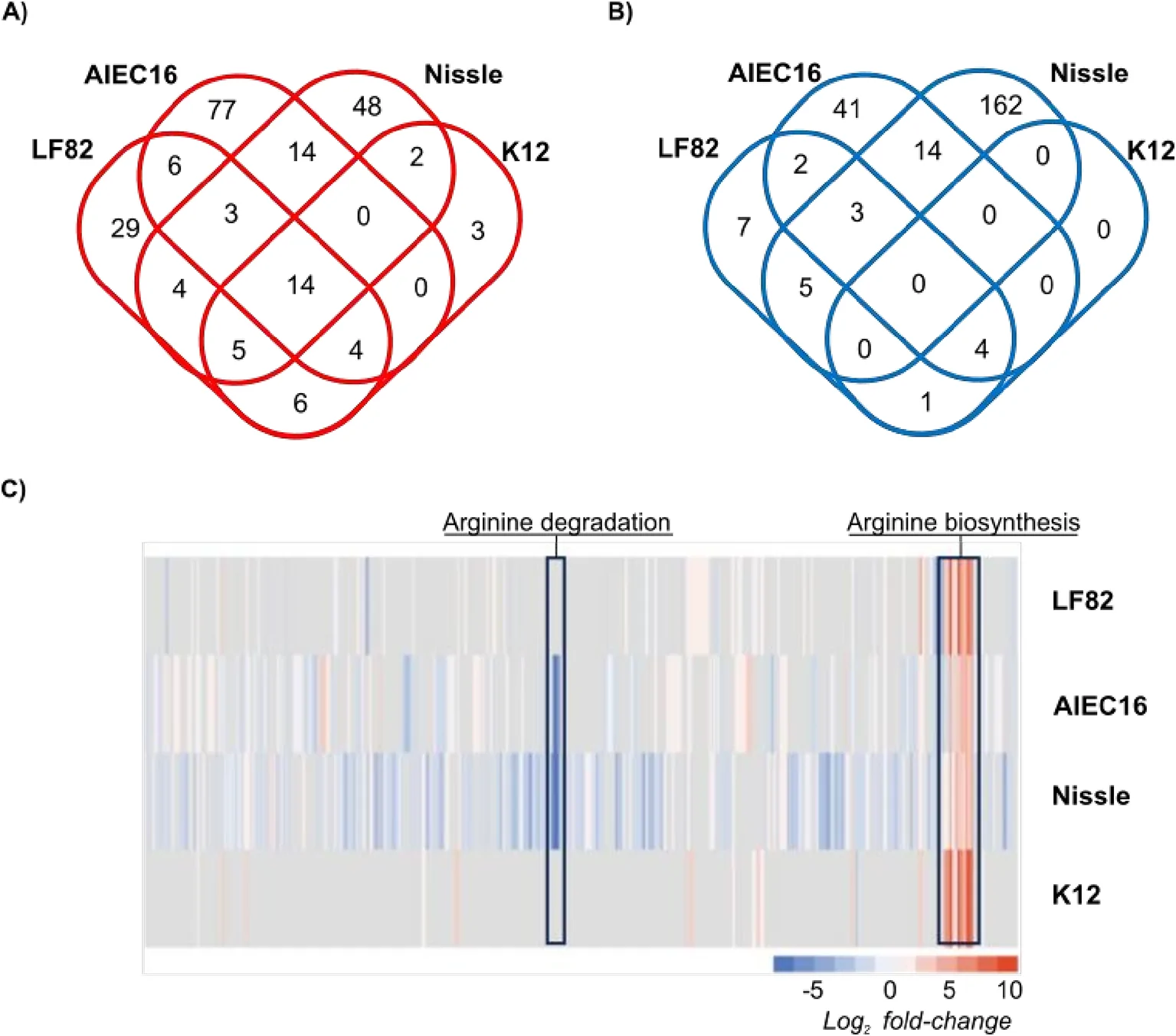
Comparative transcriptomics between Δ*argR* mutants and WT. **(A)** Venn diagram of upregulated genes in Δ*argR* mutants. **(B)** Venn diagram of downregulated genes in Δ*argR* mutants. **(C)** Heatmap of the log2 fold change values obtained in the differentially expressed genes.

In LF82, most of the genes downregulated are involved in arginine degradation and putrescine synthesis (**Figure 4**, category E), but also genes involved in the glyoxylate cycle and type-1 fimbriae (*fimA*). Most of the genes upregulated are involved in amino acid transport and metabolism (category E) (**Figure 4**). As expected, most of these genes were related to arginine, glutamine and histidine biosynthesis and transport, indicating that they are repressed by ArgR. Moreover, several motility genes involved in the flagellar structure (*fliD*), motor (*motAB*) and chemotaxis (*tar*) were found upregulated in absence of ArgR (**Supplementary Tables 4**; **7**), which correlates with the increased motility observed in the absence of *argR* (**Figure 1A**).

**Figure 4:**
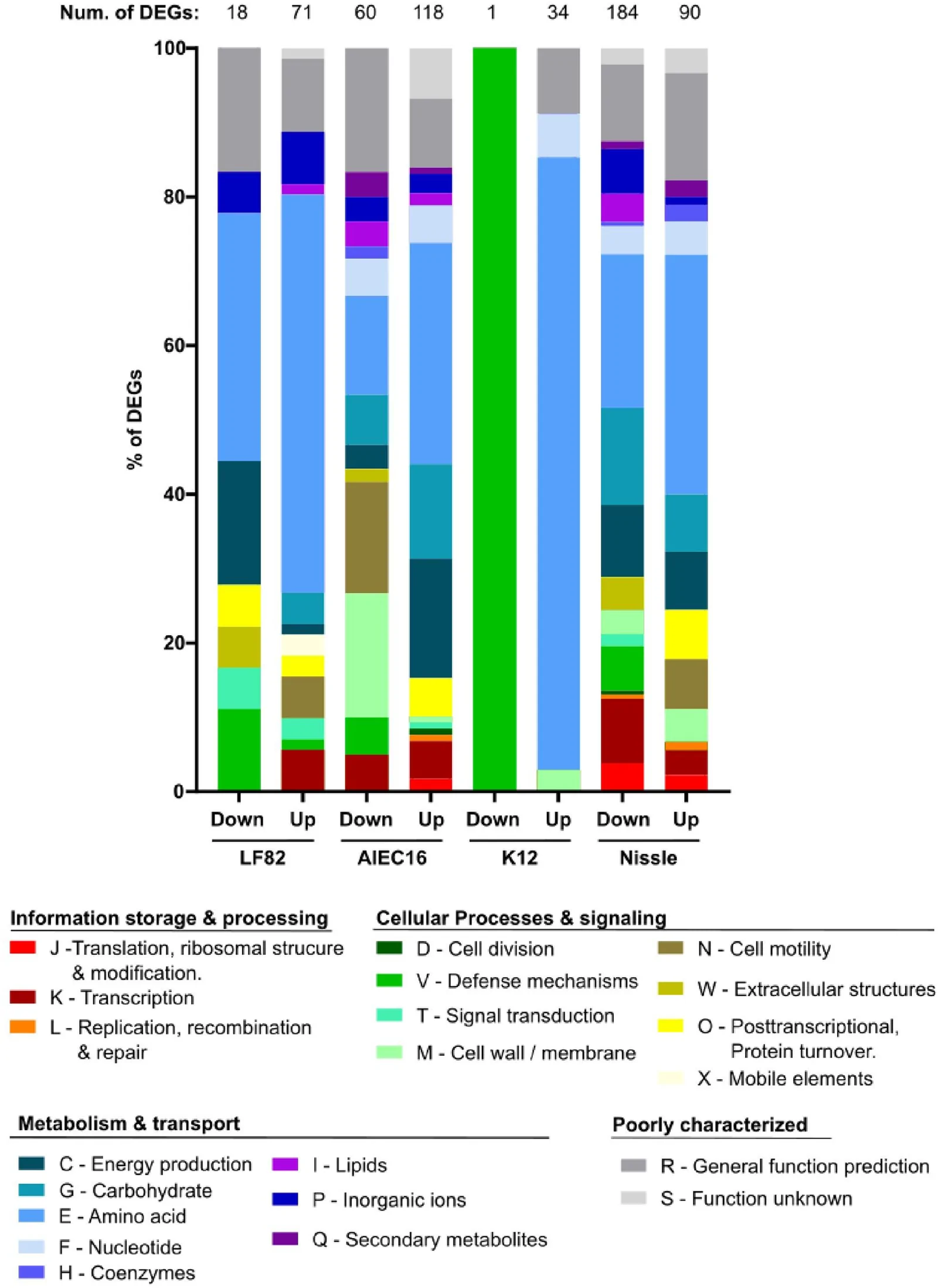
Differentially expressed genes between Δ*argR* mutants and WT classified in COGs categories. Genes indicated as up- or down-regulated correspond to expression changes in the Δ*argR* mutant compared with the wild-type strain.

In AIEC16, the absence of ArgR lead to the downregulation of the genes involved in the production of the enterobacterial common antigen (ECA) (**Supplementary Table 7**), which seem to play an important role in the survival in host environment and resistance to bile salts, acetic acid and antibiotics (Mitchell et al., 2018; Bennett and Mitchell, 2025), as well as some genes involved in LPS production (**Figure 4**, category M). Genes involved in motility (*fliA* – σ^28^ subunit – and other structural, and chemotaxis genes) were also downregulated in ArgR mutants, correlated with the motility decrease observed (**Figure 1A**). Moreover, genes involved in the production of type-1 fimbriae (*fimA*) and curli (*csgG*) are downregulated in the absence of ArgR, probably promoting the reduction of biofilm observed in **Figure 1B**. The expression of *rdcB*, which controls the activity of DgcE diguanylate cyclased, was also found downregulated.

Most of the genes upregulated in AIEC16 are involved in metabolic pathways (**Figure 4**), with the majority belonging to arginine biosynthesis and transport, but also histidine and lysine, putrescine production, and the acid-resistance modules Adi and GAD (category E), although no differences in acid survival were detected (**Supplementary Figure 1**). In addition, several genes involved in energy production – particularly anaerobic fermentation and nitrite reductase (category C) – together with carbohydrate degradation pathways such as the hexuronate system (category G), which reflects the ability to catabolize mucin-derived glycans, were also upregulated (**Figure 4****;** **Supplementary Table 5**).

In K-12, ArgR regulates a few genes (35), as previously indicated (**Table 2**), and most of them are found upregulated in the *argR* mutant strain, suggesting that they are repressed by ArgR. Of those, 28 belong to amino acid metabolism pathways (category E). Apart from genes with unknown functions, the gene *amiC*—an amidase essential for cellular division—was also found to be upregulated by ArgR (category M). The only gene downregulated in the *argR* mutant strain was *dps*, which is involved with bacterial DNA protection during stationary phase (category V, **Figure 4**).

In Nissle, as in the other strains, the genes related to amino acid metabolism (category E) were the ones mostly affected by ArgR deletion, being the arginine biosynthesis and transport genes upregulated. However, in this case most of the category E genes were downregulated in the mutant strain, suggesting that ArgR acts as an activator (**Figure 4**). These genes were related to amino acid biosynthesis (such as valine, leucine and lysine) and degradation (proline and serine). Genes related with energy production, carbohydrate transport and metabolism, defense mechanisms (siderophore yersiniabactin) and extracellular structures (type I fimbriae) were activated by ArgR (**Figure 4**, **Supplementary Table 7**). Otherwise, ArgR acted as a repressor of genes falling into the category N (motility) and O category genes (posttranscriptional modification and chaperones). Four genes that were upregulated in the mutant strains were related to the glutamate decarboxylase acid resistance system (GAD). However, no differences were observed in the acid resistance assays.

## 4 Discussion

To determine the involvement of arginine biosynthesis genes in the AIEC phenotype, Δ*argR* mutants of the AIEC LF82, AIEC16, *E. coli* Nissle 1917 and *E. coli* K-12 C600 strains were generated. As expected, the absence of ArgR lead to the overexpression of several genes involved in arginine biosynthesis, what was corroborated by RT-qPCR and mRNA sequencing. This is consistent with the well-established role of ArgR as a repressor of the *arg* regulon and the arginine transport systems *artPIQM* (Caldara et al., 2006). Conversely, genes of the arginine succinyltransferase pathway (*astA-E*), which requires ArgR for full induction under nitrogen-limiting conditions, where downregulated in the Δ*argR* mutants (**Supplementary Table 4**), in agreement with previous observations.

ArgR deletion affected motility in a strain-specific manner (**Figure 1A**, **Table 3**). No motility differences were observed in the non-AIEC strains, whereas the two AIEC strains responded in opposite directions: motility increased in LF82 Δ*argR* but decrease in AIEC16 Δ*argR*. These phenotypes were consistent with the transcriptional profiles of flagellar genes (**Figure 4**, **Supplementary Tables 4**; **5**). AIEC16 Δ*argR* exhibited downregulation of the flagellar sigma factor *fliA*, which is required for expression of late flagellar genes, while LF82 Δ*argR* showed upregulation of genes encoding components of the flagellar rotor (*motA* and *motB*) and the flagellar cap (*fliD)*. Previous studies show that ArgR can influence motility through different regulatory routes depending on the bacterial background. In *E. coli* K-12, ArgR binds the master regulator *flhDC* promoter and represses motility (Masui et al., 2026); in *Aeromonas veronii*, ArgR enhances motility by activating *flrBC* and repressing diguanylate cyclases (Wang et al., 2024); and in *Pseudomonas putida*, ArgR affects motility indirectly through its impact on arginine metabolism, cyclic-di-guanosine monophosphate (c-di-GMP) turnover, and stationary-phase regulators such as RpoS (Barrientos-Moreno et al., 2022). Notably, the described ArgR-binding site in the *flhDC* regulatory region (Masui et al., 2026), is fully conserved in both LF82 and AIEC16 (**Supplementary Figure 2**). Instead, comparison of the promoter region between both strains, revealed a nucleotide substitution located between the described -35 and -10 elements (**Supplementary Figure 2**). Such variations in promoter architecture may influence basal promoter activity and/or the transcriptional response to ArgR, potentially contributing to the strain-specific effect observed on flagellar genes expression and motility. Moreover, we cannot exclude indirect effects, such as differential interactions with global regulators such as c-di-GMP or alternative sigma factors.

**Table 3:** Summary of the results obtained from bacterial physiology assays comparing WT and Δ*argR* strains, and their relationship with the gene expression differences detected in the comparative transcriptomics.

Feature	Phenotype			Related DEGs		
	LF82 Δ*argR*	AIEC16 Δ*argR*	Nissle Δ*argR*	LF82 Δ*argR*	AIEC16 Δ*argR*	Nissle Δ*argR*
Motility	**↑**	**↓**	**-**	***pdeH, motA, motB, fliD, tar***	*flgL, motB, LF82_RS09990, fliD, cheZ, fliA, ycgR, cheA, tar*	***fliP, flgJ, flgN, fliO, fliE, yneE*,** *dppA, mglB, malE*
Adhesion to I-407	**-**	**-**	**-**	*fimA*	*fimA*	*fimA, fimC, fimD, fimF, fimG, fimH*, *fimI*
Invasion of I-407	**↑**	**-**	**↑**	***motA, motB, fliD***	*flgL, motB, fliA fliD, cheA, cheZ, ycgR*	***fliP, flgJ, flgN, fliO, fliE, yneE***
Survival/replication in J774	**-**	**-**	**-**	***gadW***	***gadA, gadB, gadC, gadE, adiY, ymdF, ygiW*** *, ybtX, ybtS, ybtP, ybtE, irp1, irp2, fyuA*	***gadA, gadB, gadC, gadE*** *, ychH, bsmA*
Acid resistance	**-**	**-**	**-**	***gadW***	***gadA, gadB, gadC, gadE, adiY, ymdF, ygiW***	***gadA, gadB, gadC, gadE*** *, ychH, bsmA*
Biofilm formation	**-**	**↓**	**-**	***-***	*csgG, tabA, rdcB*, ***ygiW****, ymdF, rfbA*	*tabA, ychH, bsmA, nanA*

The overexpressed genes in the Δ*argR* strains are highlighted in bold. DEG, differentially expressed gene.

Flagellin is considered a pathogen-associated molecular pattern recognized by the innate immune receptors the Toll-like Receptor 5 (TLR5) and the NOD-like receptor 4 (NLRC4), that activates the immune response. In a study performed with the LF82 strain, flagellar expression was found to be involved in the inflammatory process, exacerbating colitis in an animal model. Furthermore, under intestinal conditions, AIEC strains were able to induce flagellar expression, which was not induced in other *E. coli* strains. This provides an advantage for crossing the mucus layer and reaching the epithelial surface (Sevrin et al., 2020). Thus, strain-specific rewiring of regulatory networks may explain the opposite motility responses to ArgR deletion in AIEC16 and LF82.

Biofilm formation was altered only in the AIEC16 Δ*argR* strain, showing a significant decrease compared to their WT counterpart (**Figure 1B**, **Table 3**). This phenotype correlates with the downregulation of type-1 fimbriae (*fimA*) and curli (csgG). It may be argued that the second messenger c-di-GMP may be responsible for this variation, since it plays a role regulating the expression of *csgG* (Zhang et al., 2020). Supporting this, *rdcB*, which activates the diguanylate cyclase DgcE and promotes biofilm formation (Pfiffer et al., 2019), was downregulated in AIEC16 Δ*argR* but upregulated in LF82 Δ*argR*. Other factors such as decreased expression of *rfbA*, involved in extracellular polysaccharide synthesis, may also be involved to biofilm formation (Kim and Wei, 2009; Liu et al., 2021).

In *Salmonella enterica* serovar Typhimurium, free periplasmic L-arginine rapidly increases c-di-GMP levels and modulates the switch between motility and biofilm formation (Mills et al., 2015). By analogy, the altered nitrogen state in AIEC16 Δ*argR* could impair proper c-di-GMP regulation and explain the dual defect in motility and biofilm formation.

Adhesion to intestinal epithelial cells was not affected by ArgR deletion in any strain, despite downregulation of several type 1 fimbriae genes (*fimAICDFGH*) in Nissle Δ*argR* and of *fimA* in AIEC strains, which are responsible for adhesion by interacting with CEACAM6 receptors expressed in intestinal epithelial cells (Barnich and Darfeuille-Michaud, 2010) (**Figure 2A**, **Table 3**). This is consistent with the fact that Nissle carries additional fimbriae, such as S fimbriae and P fimbriae, which play a crucial role in adhesion (Sonnenborn and Schulze, 2009) and are not found in LF82 or AIEC16 (Camprubí-Font et al., 2018). Invasion increased in the LF82 Δ*argR* and Nissle Δ*argR*, despite no differences in adhesion, suggesting that ArgR-dependent metabolic state may influence specific steps in bacterial internalization, but not adhesion or intracellular survival (**Figure 2B**, **Table 3**). Some of the DEGs potentially responsible for this phenotype are related to flagellar function, which is essential for LF82 to invade epithelial cells. The flagellum facilitates adhesion through motility by enabling bacterial approach to host cells and may also play a role in coordinating the expression of invasion mechanisms (Barnich et al., 2003).

Growing evidence suggest that the amino acid L-arginine plays a relevant role in host-microbe interactions (Nüse et al., 2023). L-arginine not only serves as a universal source of carbon, nitrogen, and energy for both mammalian and microbial cells, it can act as a signalling molecule that is involved in bacterial virulence gene expression. For instance, in the presence of arginine, ArgR activates expression of the genes encoding the T3SS in Enterohemorrhagic *Escherichia coli* (Mills et al., 2015; Menezes-Garcia et al., 2020). In LF82, upregulation of genes involved in the utilization of L-serine was described under inflammatory conditions, leading to an increased fitness compared to bacteria that were unable to use serine (Kitamoto et al., 2020). In our study, genes involved in the utilization of L-serine (*tdcB, tdcC, tdcD, sdaB*, and *sdaC*) were downregulated in Nissle *ΔargR*, while *garK* gene (galactarate/glucarate degradation pathway), that provides an advantage for colonization of the inflamed intestine under dysbiosis (Rosas-Lemus et al., 2020), was found upregulated. Acid-resistance genes (*gadA, gadB, gadC, gadE, gadW* and *adiY*) (Brameyer et al., 2022), were overexpressed particularly in AIEC16 Δ*argR* and Nissle Δ*argR*, despite no significant effects were observed in survival to the acid conditions tested here (**Supplementary Figure 1**). This finding could suggest that transcriptional activation of individual components of the GAD and Adi systems may be insufficient to alter the overall acid resistance phenotype, which is known to depend on the coordinated action of multiple regulatory and physiological processes.

AIEC16 Δ*argR* also overexpressed oxidative-stress resistance genes (*ymdF* and *ygiW*), whereas yersiniabactin siderophore genes (*ybtX, ybtS, ybtP, ybtE, irp1, irp2*, and *fyuA*) were downregulated. Since yersiniabactin provides a selective advantage in iron limitation within the phagolysosome (Prudent et al., 2021), a decrease in survival inside macrophages was expected, but no differences were observed (**Figure 2C**). As these genes were downregulated in the absence of ArgR, we could hypothesize that ArgR may play an activation role, probably via global metabolic effect rather than direct binding.

One limitation of the present study is the relatively small number of strains analyzed. To capture part of the diversity within the *E. coli* species, we included two non-AIEC strains representing distinct ecological backgrounds, namely the laboratory strain C600 and the probiotic commensal strain Nissle 1917, as well as two AIEC strains: the reference strain LF82 and the clinical isolate AIEC16. Although LF82 and AIEC16 both belong to the B2 phylogroup, AIEC strains are known to be genetically heterogeneous and have been identified across multiple *E. coli* phylogroups. Therefore, additional studies including a larger collection of AIEC and non-AIEC isolates will be required to determine how broadly the phenotypes described here apply across the diversity of the pathotype.

In summary, our results indicate that ArgR plays a key role in controlling arginine metabolism across *E. coli* strains, but its deletion triggers strain-specific alterations of global regulatory networks in AIEC. Rather than acting primarily through direct regulation of virulence genes, ArgR appears to shape the metabolic state that feeds into higher-order regulators such as c-di-GMP, FNR/Nar, CRP, GadE or RpoS, leading to distinct outcomes in motility, biofilm formation, stress resistance, and invasion in different backgrounds. Further studies are needed to determine how arginine availability and ArgR activity integrate with these global pathways during AIEC colonization of the inflamed intestine.

## Data Availability

The data presented in the study are deposited in the CORA repository (Martinez-Medina et al., 2026).
