# Supplementary material for: The arginine repressor ArgR controls virulence factors of adherent-invasive Escherichia coli strains
Source: Front Cell Infect Microbiol. 2026 Oct 12;16:1941024. doi: 10.3389/fcimb.2026.1941024 (PMC13696330; doi:10.3389/fcimb.2026.1941024)
Supplement: Supplementary file 1 [file SupplementaryFile1.docx]

Supplementary Material

# 1 Supplementary Figures

**Supplementary Figure 1.** Effects of ArgR deletion on the resistance to acidic conditions. Percentage of survival was assessed after 1h incubation on Ringer at different pH conditions. Statistical significance was assessed through Mann-Whitney test. No significant differences were observed between WT and ∆*argR* strains.

**Supplementary Figure 2.** Alignment of the *flhDC* promoter region from LF82 and AIEC16. The -10 and -35 promoter elements are indicated in red, and transcription start site (+1) is shown in uppercase bold letters. The ArgR-binding site previously described for *E. coli* MG1655 (Masui et al., 2026) is indicated in blue. Red arrows indicate nucleotide polymorphisms between LF82, AIEC16 and MG1655.

# 2 Supplementary Tables

Supplementary Tables can be accessed here: <https://doi.org/10.34810/DATA3287>.

**Suppl. Table 1.** Strains and plasmids used in the bacterial mutagenesis steps.

**Suppl. Table 2.** Primers used to synthetize the linear DNA fragment used to generate ∆*argR* strains and to verify them using PCR and RT-qPCR.

**Suppl. Table 3.** General results obtained in the RNA sequencing (sample IDs, RNA concentration measured in Qubit, number of reads retained after quality filtering, and number and proportion of reads mapped uniquely against the reference genome). * Percentages calculated over the total number of reads used as input in each step.

**Suppl. Table 4.** Differentially expressed genes upregulated in Δ*argR* strains compared with WT, putatively repressed by ArgR. N.D.: Not Determined

**Suppl. Table 5.** Differentially expressed genes downregulated in Δ*argR* strains compared with WT, putatively activated by ArgR. N.D.: Not Determined

**Suppl. Table 6.** DEGs involved in arginine biosynthesis, transport or degradation. Under: Underexpressed in Δ*argR*; Over: Overexpressed in Δ*argR*; A: AIEC16 Δ*argR* vs AIEC16; K: K-12 Δ*argR* vs K-12; L: LF82 Δ*argR* vs LF82; N: Nissle Δ*argR* vs Nissle.

**Suppl. Table 7.** DEGs involved in bacterial pathogenicity. For each gene, FPKM and log2 fold change values obtained in the comparative transcriptomics are shown. Moreover, details regarding gene function and the comparison where the differences of expression were detected are depicted.
